# IL‐8 induces transdifferentiation of mature hepatocytes toward the cholangiocyte phenotype

**DOI:** 10.1002/2211-5463.12750

**Published:** 2019-11-07

**Authors:** Tokio Sasaki, Yuji Suzuki, Keisuke Kakisaka, Ting Wang, Kazuyuki Ishida, Akiko Suzuki, Hiroaki Abe, Tamotsu Sugai, Yasuhiro Takikawa

**Affiliations:** ^1^ Division of Hepatology Department of Internal Medicine Iwate Medical University School of Medicine Morioka Japan; ^2^ Department of Molecular Diagnostic Pathology Iwate Medical University School of Medicine Morioka Japan

**Keywords:** cholangiocyte, interleukin‐8, mature hepatocyte, Sry HMG box protein 9, transdifferentiation

## Abstract

The adult mammalian liver exhibits a remarkable regenerative capacity, with different modes of regeneration according to the type and extent of injury. Hepatocyte–cholangiocyte biphenotypic liver progenitor cell populations appear under conditions of excessive injury. It has been reported that mature hepatocytes can transdifferentiate toward a cholangiocyte phenotype and be a cellular source of progenitor cell populations. Here, we determined that among various plasma cytokines, interleukin (IL)‐8 levels were significantly elevated in acute liver failure and severe acute liver injury patients. *In vitro* assays revealed that administration of IL‐8 homologues increases the expression of Sry HMG box protein 9 (SOX9). In liver biopsies of acute liver injury patients, we observed the appearance of SOX9‐positive biphenotypic hepatocytes accompanied by elevation of plasma IL‐8 levels. Our results suggest that IL‐8 regulates the phenotypic conversion of mature hepatocytes toward a cholangiocyte phenotype.

## Abstract 2

Interleukin (IL)‐8 treated mouse mature hepatocytes expressed Sry HMG box protein 9 (SOX9), a bile duct‐associated marker. In liver biopsies of acute liver injury patients, SOX9‐positive biphenotypic hepatocytes appeared in the liver parenchyma which is accompanied by elevation of plasma IL‐8 levels. Our results suggest that IL‐8 regulates the phenotypic conversion of mature hepatocytes toward a cholangiocyte phenotype.

## Graphical abstract

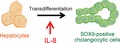

**Abbreviations**
ALF: acute liver failure
ALI: acute liver injury
DAPT: *N*‐[*N*‐(3,5‐difluorophenacetyl‐L‐alanyl)]‐S‐phenylglycine t‐butyl Ester
DRs: ductular reactions
EpCAM: epithelial cell adhesion molecule
IL: interleukin
LPCs: liver stem/progenitor cells
MIP: macrophage inflammatory protein
PT‐INR: prothrombin time‐international normalized ratio
qRT‐PCR: quantitative real‐time PCR
SLI: severe acute liver injury
SOX9: Sry HMG box protein 9

Tissues that makeup organs are diverse in the dynamics of their constituent cells as well as in their structures and functions 1. Each organ can maintain homeostasis via cellular replication of differentiated cells or differentiation from stem cell populations 2. The adult mammalian liver has a remarkable regenerative capacity, with different modes of regeneration according to the type and extent of injury. Specifically, under physiological conditions, new hepatocytes arise via replication of preexisting hepatocytes during the homeostatic renewal of the liver 3, 4, 5. In contrast, when the proliferation of hepatocytes is impaired as a result of acute or chronic liver injury—such as acute liver failure (ALF), chronic viral hepatitis, and nonalcoholic fatty liver disease 6, 7—the liver stem/progenitor cells (LPCs), which are cells with intermediate hepatocyte–cholangiocyte phenotype, emerge and expand in the liver parenchyma 8, 9, 10. Histologically, the LPCs expand in the parenchyma forming duct‐like structures, which are known as ductular reactions (DRs) 11. DRs may arise from the preexisting biliary epithelium 12, or, as recent evidence shows, from mature hepatocytes that transdifferentiate into a cholangiocyte phenotype 13, 14, 15.

Sry HMG box protein 9 (SOX9) is a transcription factor that plays pivotal roles during embryonic development of several tissues, such as the testis, pancreas, bile duct, lung, heart, and central nervous system, as well as chondrocytes 16, 17, 18, 19. SOX9 is one of the earliest biliary markers that regulate bile duct development 20. Recently, it has been suggested that a subpopulation of periportal hepatocytes of the normal liver expresses SOX9 and proliferates during chronic liver injury to contribute to the regeneration of the liver 21.

During ALF, biliary phenotypic cells expand into the liver parenchyma, which is accompanied by a drastic decrease in the number of preexisting hepatocytes. In our previous study, we showed that ALF patient plasma promotes the proliferation of LPCs *in vitro* 22. Additionally, using our murine hepatectomy model that involves reactions to mechanical injury with inflammation, we have demonstrated that bile duct‐like structures emerge in the injury front 23. In this present study, we attempted to identify cytokines in the plasma of ALF and severe acute liver injury (SLI) patients that are responsible for the phenotypic conversion of mature hepatocytes. Our findings may provide insights into the cellular plasticity of hepatocytes in response to inflammation in the injured liver.

## Materials and methods

### Measurement of cytokines in human plasma

From June 2012 to December 2014, the plasma cytokine levels of 37 ALF and SLI patients were determined on admission using a Bio‐Plex Pro human cytokine 27‐plex assay (Bio‐Rad, Hercules, CA, USA). Of the 27 types of cytokines, we examined interleukin (IL)‐8 levels along with those of eight other inflammatory cytokines for which the normal range was previously validated in healthy volunteers 24. Serum CXCL1 and CXCL2 levels were examined using commercially available ELISA kits (ab190805 and ab184862, respectively; Abcam, Cambridge, UK). SLI is defined as an acute hepatic illness of < 26 weeks with a prothrombin time‐international normalized ratio (PT‐INR) of ≥ 1.5, along with absence of hepatic encephalopathy in a patient without preexisting chronic liver disease 25. ALF is defined as an acute hepatic illness of < 26 weeks with a PT‐INR of ≥ 1.5 and any degree of mental alteration (encephalopathy) in a patient without preexisting chronic liver disease 26 (Table S1). Characteristics of the patients are summarized in Table 1. All protocols reported in this study were approved by the institutional review board of Iwate Medical University (approval number: H20‐36). Informed written consent was obtained from all participants and the present study was designed and conducted in accordance with relevant guidelines and regulations of the ethical principles for medical research involving human subjects defined by the WMA Declaration of Helsinki.

**Table 1:** Clinical characteristics of ALF and SLI patients. Data are presented as *n* (%) or medians (interquartile range). AST, aspartate aminotransferase; ALT, alanine aminotransferase.

	*n* = 37
Age, median (range), years	65 (51.0–72.0)
Male, *n* (%)	17 (45.9)
Etiology, *n* (%)	
Autoimmune hepatitis	7 (18.9)
Drug	7 (18.9)
Hepatitis B virus	6 (16.2)
Hepatitis A virus	2 (5.4)
Hepatitis E virus	2 (5.4)
Other	4 (10.8)
Unknown	9 (24.3)
Survived without transplant, *n* (%)	30 (81.1)
AST, median (range), U·L^−1^	1489 (458–1858)
ALT, median (range), U·L^−1^	1480 (671–2616)
Total bilirubin, median (range), mg·dL^−1^	11.5 (3.7–20.4)
PT‐INR, median (range)	1.66 (1.47–2.29)

### Cell lines and culture

AML12 mouse mature hepatocytes (ATCC, Manassas, VA, USA), a cell line established from a human TGF‐α transgenic mouse, was maintained in Dulbecco's Modified Eagle's Medium (DMEM)/Ham's F12 media containing 10% fetal bovine serum (FBS) supplemented with 5 µg·mL^−1^ insulin, 5 µg·mL^−1^ transferrin, 5 ng·mL^−1^ selenium, and 40 ng·mL^−1^ dexamethasone. 603B mouse cholangiocytes, a cell line established from a mouse transfected with a thermosensitive mutant SV40 T antigen, was maintained in DMEM medium containing 10% FBS. The 603B cell line was kindly provided by Y. Ueno of the University of Yamagata 27. An epithelial cell adhesion molecule (EpCAM)‐positive liver progenitor cell line from a 3,5‐diethoxycarbonyl‐1,4‐dihydrocollidine‐fed adult mouse was maintained in Williams' medium E (Thermo Fisher Scientific, Waltham, MA, USA) containing 10% FBS, 10 mm nicotinamide, 2 mm l‐glutamine, 0.2 mm ascorbic acid, 20 mm HEPES (pH 7.5), 1 mm sodium pyruvate, 17.6 mm NaHCO3, 14 mm glucose, 100 nm dexamethasone, 50 µg·mL^−1^ gentamicin, 1% insulin‐transferrin‐selenium‐ethanolamine (Thermo Fisher Scientific), 10 ng·mL^−1^ human EGF, and 10 ng·mL^−1^ human HGF. LPCs were kindly provided by A. Miyajima and M. Tanaka of the University of Tokyo 28.

### Isolation and culture of primary mouse hepatocytes

Primary mouse hepatocytes were isolated using the digitonin‐collagenase perfusion method 29. C57BL/6J mice were anesthetized by inhalation of isoflurane (2.5% v/v) and mouse livers were initially perfused through the portal vein with 12 mL of liver perfusion medium (Thermo Fisher Scientific). Liver perfusion medium containing 4 mg·mL^−1^ digitonin (Merck Millipore, Burlington, MA, USA) was perfused until a regularly scattered periportal discoloration was observed (Fig. S1). Next, 40 mL of HEPES buffer without magnesium containing 25 mm HEPES (pH 7.4) and 0.6 mg·mL^−1^ type IV collagenase (Worthington Biochemical, Lakewood, NJ, USA) was infused via the portal vein. The liver was removed and gently agitated in HEPES buffer containing 25 mm HEPES and 2 mg·mL^−1^ bovine serum albumin. After filtering the digested liver tissue, the solution containing hepatocytes was centrifuged at 40 ***g*** for 2 min (three times) and the cells were resuspended in Waymouth medium (Thermo Fisher Scientific) containing 10% FBS, 0.1 µm insulin, and 0.1 µm dexamethasone. For flow cytometric analysis of hepatocyte purity, cells were fixed with 4% paraformaldehyde followed by permeabilizing using 0.2% Triton X‐100 (Sigma‐Aldrich, St. Louis, MO, USA). Cells were then incubated with a rabbit anti‐albumin antibody (1 : 100; ab207327; Abcam) or a rabbit IgG isotype control antibody (ab172730; Abcam) for 30 min at 4 °C. After several washes, the cells were stained with goat anti‐rabbit IgG Alexa Fluoro 488 secondary antibody (1 : 2000, ab150077; Abcam) for 30 min at 4 °C. Flow cytometry was performed using a BD FACSCanto II system (BD Biosciences, San Diego, CA, USA). More than 5000 cells were counted for each sample. The obtained data were analyzed with facsdiva software (BD Biosciences). All animal experiments were approved by the Iwate Medical University Ethical Committee for Animal Experiment Regulation (approval number: 28‐037).

### Cell proliferation assay

AML12 cells, LPCs, and 603B cells were placed in 96‐well plates at a seeding density of 1.0 × 10^4^ cells/well, cultured for 16 h, and then cultured for an additional 8 h in the absence or presence of recombinant IL‐8 homologues, keratinocyto‐derived chemokine (KC), or macrophage inflammatory protein (MIP)‐2 (American Research Products, Waltham, MA, USA), ranging from 100 pg·mL^−1^ to 10 ng·mL^−1^. Eight hours after addition of KC or MIP‐2, the number of viable cells was counted using Cell Count Reagent SF (Nacalai Tesque, Kyoto, Japan) 30. Light absorbance was measured at 450 nm with a microscope photometer (Immuno Mini NJ‐2300; InterMed, Tokyo, Japan).

### Western blotting

For western blotting analysis, AML12 cells and LPCs were placed in 6‐well plates at a seeding density of 1.0 × 10^6^ cells/well, cultured for 24 h, and then cultured for an additional 8 h in the absence or presence of KC and MIP‐2, ranging from 100 pg·mL^−1^ to 10 ng·mL^−1^. Cells were lysed for 30 min on ice with lysis buffer [50 mm per liter Tris/HCl (pH 7.4), 1% Nonidet P‐40, 0.25% sodium deoxycholate, 150 mm NaCl, 1 mm EDTA, 1 mm phenylmethylsulfonyl fluoride, 1 μg·mL^−1^ aprotinin, 1 μg·mL^−1^ leupeptin, 1 μg·mL^−1^ pepstatin, 1 m Na_3_VO_4_, and 1 mm NaF] 31. After centrifugation at 13 000 ***g*** for 10 min, the protein concentration in the supernatant was measured using the Bradford reagent (Bio‐Rad, Tokyo, Japan). The supernatant protein was denatured by boiling for 10 min. Thirty micrograms of protein from each sample was resolved in a sodium dodecyl sulfate/polyacrylamide gel electrophoresis gradient gel (4–12%) and then transferred onto nitrocellulose membranes. Blocking was carried out using 5% nonfat dry milk in Tris‐buffered saline (20 mm Tris and 150 mm NaCl, pH 7.4) with 0.1% Tween 20 for 2 h at 20–24 °C. Anti‐cyclin D1 antibody (1 : 1000; sc‐717; Santa Cruz Biotechnology, Dallas, TX, USA) and anti‐β‐actin antibody (1 : 1000; sc‐1616; Santa Cruz Biotechnology) were diluted in blocking solution and incubated overnight at 4 °C. To detect antigen–antibody complexes, peroxidase‐conjugated secondary antibodies [1 : 10 000; sc‐2357 (anti‐rabbit), sc‐2354 (anti‐goat); Santa Cruz Biotechnology] were diluted in blocking solution and incubated for 2 h at room temperature. Immune complexes were detected using chemiluminescence with the ECL Prime Western Blotting Reagent (GE Healthcare, Tokyo, Japan).

### RNA preparation and reverse transcription PCR

Total RNA was extracted using RNeasy Mini kit (Qiagen, Tokyo, Japan) according to the manufacturer's protocol. First‐strand cDNA was synthesized from 100 ng of total RNA using the High‐Capacity cDNA RT Kit (Applied Biosystems, Foster City, CA, USA). For semi‐quantitative RT‐PCR analysis, PCR amplification was performed with Takara Ex Taq (Takara Bio, Kusatsu, Japan). PCR was carried out using a Takara PCR Thermal Cycler Dice (Takara Bio). PCR conditions were as follows: initial activation of the Taq‐DNA‐Polymerase at 94 °C for 5 min, denaturation at 94 °C for 30 s, annealing at 60 °C for 30 s, and extension at 72 °C for 30 s. For normalization, mouse glyceraldehyde‐3‐phosphate dehydrogenase mRNA was simultaneously amplified. Amplified PCR products were separated by agarose gel electrophoresis and stained with ethidium bromide.

For quantitative real‐time PCR analysis (qRT‐PCR), AML12 cells were placed in 6‐well plates at a seeding density of 1.5 × 10^5^ cells/well and then cultured for 120 h in the absence or presence of 10 ng·mL^−1^ of KC, MIP‐2, or tumor necrosis factor (TNF)‐α (Peprotech, Rocky Hill, NJ, USA). Primary hepatocytes were placed in type I collagen‐coated 6‐well plates at a seeding density of 2.0 × 10^5^ cells/well and then cultured for 72 h in the absence or presence of 10 ng·mL^−1^ of either KC or MIP‐2. Interaction between Notch and IL‐8 homologue signaling pathways was tested by applying 10 µm γ‐secretase inhibitor *N*‐[*N*‐(3,5‐difluorophenacetyl‐l‐alanyl)]‐*S*‐phenylglycine t‐butyl Ester (DAPT), an inhibitor of Notch signaling (D5942; Sigma‐Aldrich). For qRT‐PCR, FastStart SYBR Green Master (Roche Diagnostics, Mannheim, Germany) was used. All real‐time PCR procedures were performed using a Light‐Cycler (Roche Diagnostics). RNA expression was normalized with the housekeeping 18s rRNA gene. Primer sequences are listed in Tables S2 and S3.

### Immunofluorescence labeling of cells and fluorescence microscopy

AML12 cells were placed in 6‐well plates at a seeding density of 1.5 × 10^5^ cells/well and then cultured for 120 h in the absence or presence of 10 ng·mL^−1^ of KC, MIP‐2, or TNF‐α (Peprotech). Primary hepatocytes were placed in type I collagen‐coated 6‐well plates at a seeding density of 2.0 × 10^5^ cells/well and then cultured for 72 h in the absence or presence of 10 ng·mL^−1^ of either KC or MIP‐2. Interaction between Notch and IL‐8 homologue signaling pathways were tested by applying 10 µm DAPT. The detailed protocol of medium change is indicated in the figure legends. Cells were grown in slides and fixed with 4% paraformaldehyde for 10 min at room temperature. Cells were then washed in PBS three times and incubated with PBS containing 0.2% Triton X‐100 (Sigma‐Aldrich) for 20 min at room temperature. Next, cells were washed in PBS three times, blocked with PBS containing 5% fetal bovine serum and 0.2% Triton X‐100 for 60 min at room temperature, and then incubated with diluted rabbit anti‐SOX9 antibody (1 : 236; ab185966; Abcam), anti‐EpCAM antibody (1 : 400; ab71916; Abcam), and anti‐CK‐19 antibody (1 : 400; ab52625; Abcam) overnight at 4 °C. After several washes, the slides were incubated with goat anti‐rabbit antibody (1 : 2000; A27039; Invitrogen, Carlsbad, CA, USA) for 2 h at room temperature in the dark. The slides were washed three times in PBS and stained with 4′,6‐diamidino‐2‐phenylindole (DAPI) to visualize the nucleus. Finally, the slides were washed, mounted with glycerin, and analyzed using an EVOS fluorescence microscope (Thermo Fisher Scientific).

### Immunohistochemistry of liver biopsy

Paraformaldehyde‐fixed paraffin‐embedded (FFPE) (4%) liver tissue blocks were cut into 3‐µm sections. For colorimetric immunohistochemical staining, rabbit anti‐SOX9 antibody (1 : 4000; AB‐5535; Merck Millipore) was used as a primary antibody. After incubation with primary antibody, the FFPE sections were incubated with peroxidase‐labeled anti‐rabbit antibodies (Histofine Simple Stain Max‐PO Kit; Nichirei, Tokyo, Japan). In stained biopsy sections, five portal tract regions were selected per slide at 300× magnification, and then SOX9‐positive hepatocytes and SOX9‐positive cholangiocytes within an area of 0.2 mm^2^ per portal tract were counted. For immunofluorescence detection, the following primary antibodies were used on the FFPE sections: rabbit anti‐SOX9 antibody (1 : 4000; AB‐5535; Merck Millipore), rabbit anti‐EpCAM antibody (1 : 4000; ab71916; Abcam), rabbit anti‐CK19 antibody (1 : 300; ab15463; Abcam), mouse anti‐HNF4α antibody (1 : 75; sc‐374229; Santa Cruz Biotechnology), and mouse anti‐Hep‐par1 antibody (1 : 400; V2341‐20UG; NSJ Bioreagents, San Diego, CA, USA). The anti‐SOX9 primary antibodies were detected with anti‐rabbit Alexa Fluor 596 (ab150076; Abcam). The anti‐HNF4α and anti‐Hep‐par1 antibodies were incubated with peroxidase‐labeled anti‐rabbit secondary antibody (Histofine Simple Stain Max‐PO Kit; Nichirei). The bound secondary antibody was detected with TSA Fluorescein (SAT701001EA; Perkin Elmer Life Sciences, Waltham, MA, USA). Nuclei were counterstained with DAPI. Images were obtained using laser confocal microscopy (Nikon, Tokyo, Japan).

### Statistical analysis

Data are expressed as the mean ± SEM. Demographic data of patients are presented as median and interquartile range or *n* (%) for continuous and categorical variables. Statistical analyses, including Student's unpaired two‐tailed *t*‐test and Mann–Whitney *U*‐test, were conducted using graphpad prism version 7 (GraphPad Software, San Diego, CA, USA). Differences were considered significant when the *P*‐value was < 0.05.

## Results

### Serum IL‐8 levels in ALF and SLI patients are significantly elevated

**Figure 1:**
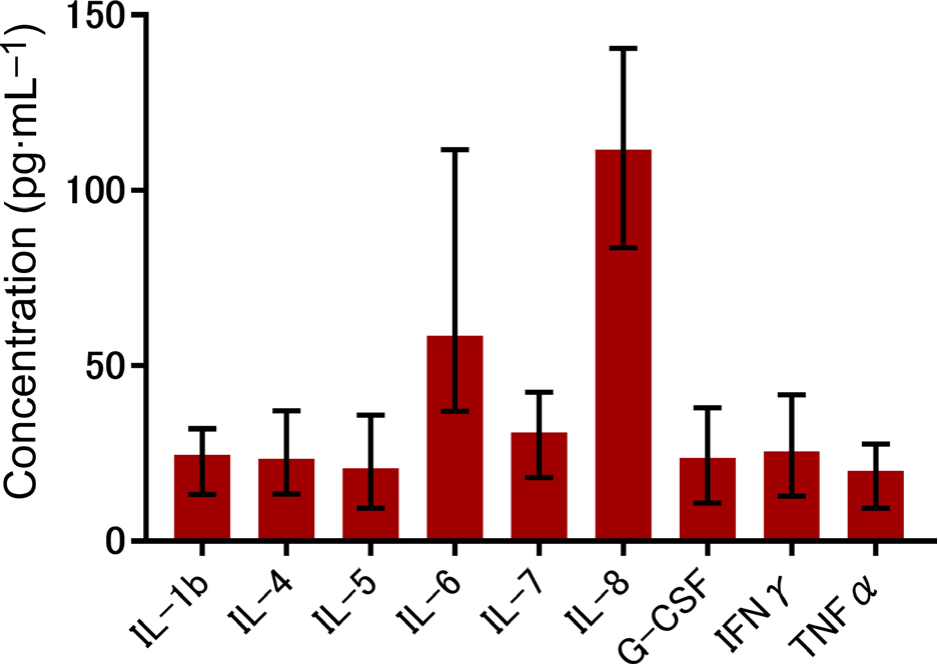
Serum cytokine levels of ALF and SLI patients. Results are presented as median and interquartile range.

Figure 1 and Table S4 show serum cytokine levels of ALF and SLI patients; IL‐8 levels were the highest among the measured cytokines. It has been reported that serum IL‐8 levels are elevated following *N*‐acetyl‐*p*‐aminophenol overdose and are predictive of hepatocellular damage 32. In addition, IL‐8 levels are significantly elevated in chronic liver disease patients depending on the cirrhosis stage 33. Therefore, we focused on the effects of IL‐8 on mouse mature hepatocytes in subsequent analyses.

### IL‐8 homologues suppress proliferation of mouse mature hepatocytes

**Figure 2:**
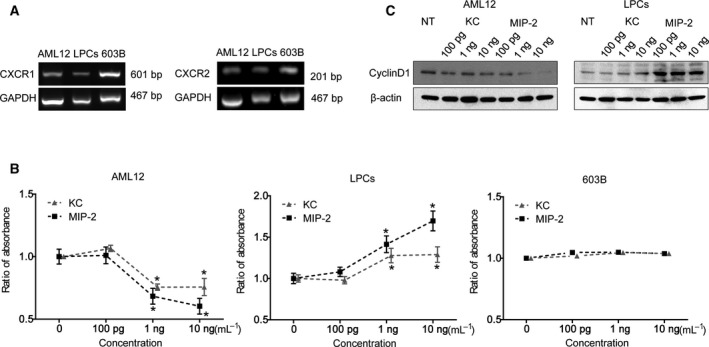
Effects of KC and MIP‐2 on the proliferative activity of mouse liver cell lines. (A) *Cxcr1* and *Cxcr2* mRNA levels in AML12, LPCs, and 603B cells. Glyceraldehyde 3‐phosphate dehydrogenase (*Gapdh*) mRNA was used as the internal control. (B) Cell viability of AML12, LPCs, and 603B cells were estimated in response to administration with KC or MIP‐2 (100 pg·mL^−1^, 1 ng·mL^−1^, or 10 ng·mL^−1^). Student's unpaired *t*‐test was performed to compare treated and control samples. Results are presented as mean ± SEM (*n* = 3), *P < 0.05. (C) Western blotting analysis was performed using cell lysates of AML12 and LPCs and antibodies specific for cyclin D1 as indicated. Both AML12 and LPCs were treated with KC or MIP‐2 (100 pg·mL^−1^, 1 ng·mL^−1^, or 10 ng·mL^−1^). β‐Actin was used as the internal control.

We determined whether administration of IL‐8 homologues KC and MIP‐2 affected the proliferative activity of a mature hepatocyte cell line (AML12) *in vitro*. Previous studies have proposed that human IL‐8 induces recruitment and activation of neutrophils to inflamed sites by two G‐protein‐coupled receptors: CXCR1 and CXCR2 34. The homologue of human IL‐8 is absent in the mouse genome. The chemokines KC and MIP‐2, which also bind to the CXCR1 and CXCR2 receptor, compensate as a functional homologue of IL‐8 in mice 35, 36. Therefore, we used KC and MIP‐2 in our mouse experiments instead of IL‐8. Before the proliferation assay, we verified whether the AML12, LPCs, and 603B cell lines expressed the IL‐8 receptor protein‐encoding genes *Cxcr1* and *Cxcr2* using reverse transcription PCR. We confirmed that all cell lines expressed both the *Cxcr1* and *Cxcr2* genes (Fig. 2A). The effects of IL‐8 on mouse cell proliferation were evaluated using cell proliferation assays. The administration of KC and MIP‐2 significantly suppressed the proliferation of AML12 cells (Fig. 2B, left). In contrast, KC and MIP‐2 significantly promoted the proliferation of LPCs (Fig. 2B, middle), whereas KC and MIP‐2 did not affect the proliferative activity of 603B cells (Fig. 2B, right). Incubation with KC or MIP‐2 suppressed cyclin D1 expression in AML12 cells and upregulated its expression in LPCs (Fig. 2C).

### IL‐8 homologues upregulate *Sox9* mRNA and SOX9 protein levels in immortalized mouse mature hepatocytes

**Figure 3:**
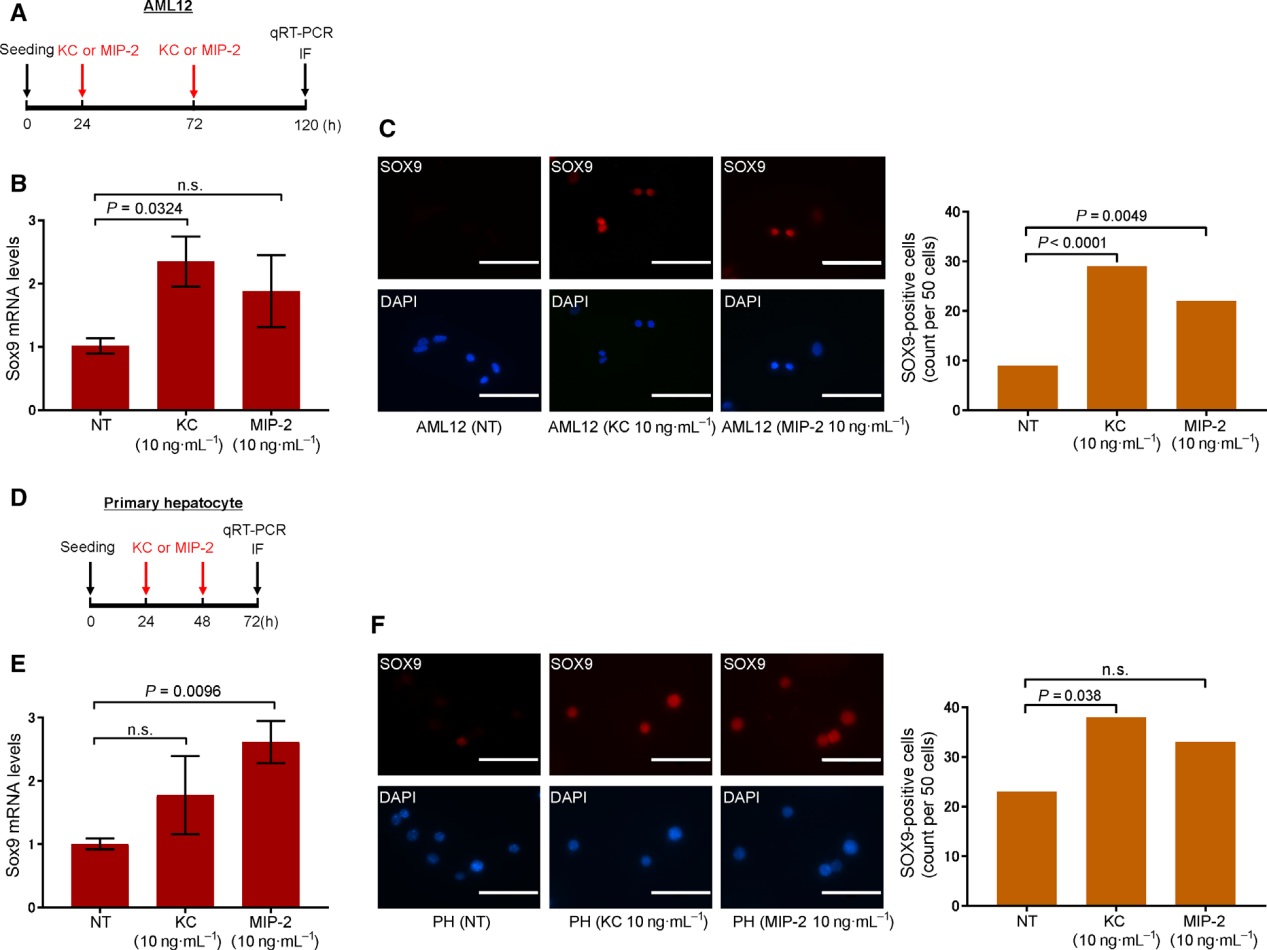
Gene expression and immunohistochemical analysis of SOX9. (A) Timeline of experimental protocols of AML12 for quantitative reverse transcription PCR (qRT‐PCR) and immunofluorescence labeling of cells. AML12 cells were seeded at 5.0 × 10^4^ cells·mL^−1^ into 6‐well plates (1.5 × 10^5^ cells/well). After incubation for 24 h, medium with 10 ng·mL^−1^ of KC or MIP‐2 was changed at 24 and 72 h. (B) *Sox9* mRNA levels in AML12 cells collected after 120 h of incubation was determined using qRT‐PCR. mRNA levels were normalized using 18s rRNA as a housekeeping gene. Student's unpaired two‐tailed *t*‐test was performed. Results are represented as mean ± SEM (*n* = 3). (C) Immunofluorescent staining of SOX9 in AML12 cells treated with or without KC and MIP‐2 after 120 h of incubation. The number of SOX9‐positive cells was quantified per 50 cells. Fisher's exact test was performed to compare treated and control samples. Scale bar, 100 μm. (D) Timeline of the experimental protocols of primary hepatocytes for qRT‐PCR and immunofluorescence labeling of cells. Primary hepatocytes were seeded at 1.0 × 10^5^ cells·mL^−1^ into 6‐well plates (2.0 × 10^5^ cells/well). After incubation for 24 h, medium with 10 ng·mL^−1^ of KC or MIP‐2 was changed at 24 and 48 h. (E) *Sox9* mRNA levels in primary hepatocytes collected after 72 h of incubation was determined using qRT‐PCR. mRNA levels were normalized using 18s rRNA as a housekeeping gene. Student's unpaired two‐tailed *t*‐test was performed. Results are represented as mean ± SEM (*n* = 3). (F) Immunofluorescent staining of SOX9 in primary hepatocytes treated with or without KC and MIP‐2 after 72 h of incubation. Primary hepatocytes were cultured in the absence or presence of 10 ng·mL^−1^ of KC or MIP‐2. The number of SOX9‐positive cells was quantified per 50 cells. Fisher's exact test was performed to compare treated and control samples. Scale bar, 100 μm. PH, primary hepatocyte.

Following the results that KC and MIP‐2 suppressed the proliferation of mature hepatocytes, we examined the effects of KC and MIP‐2 on the differentiation of these cells. It was reported that TNF‐α promotes phenotypic conversion of mature hepatocytes into cholangiocytes 37. Therefore, we hypothesized that IL‐8 induces the conversion of hepatocytes into cholangiocytic status. mRNA levels of *Sox9*, a bile duct‐associated gene and liver progenitor marker, were quantified using qRT‐PCR. Administration of 10 ng·mL^−1^ of KC for 96 h significantly upregulated *Sox9* mRNA levels in AML12 cells (*P* = 0.0324) (Fig. 3B). Administration of 10 ng·mL^−1^ of MIP‐2 for 96 h tended to upregulate *Sox9* mRNA levels, but not in a statistically significant manner. Administration of TNF‐α, a cytokine that induces phenotypic conversion of mature hepatocytes into cholangiocytes, also tended to upregulate *Sox9* mRNA levels but not in a statistically significant manner (Fig. S2). Immunofluorescence staining revealed that SOX9 protein was expressed in KC‐ or MIP‐2‐treated AML12 cells (Fig. 3C). Biliary epithelial cell markers EpCAM‐ or CK19‐protein was not detected in KC‐ or MIP‐2‐treated AML12 cells (Fig. S3A). Previous studies have proposed that Notch is the key molecule that induces transdifferentiation of mature hepatocytes into the biliary epithelial cell phenotype 15, 38, 39. Therefore, we assessed if the administration of Notch inhibitor DAPT inhibits the upregulation of *Sox9* mRNA and SOX9 protein levels caused by an IL‐8 homologue. Administration of 10 ng·mL^−1^ of KC with 10 µm of DAPT inhibited the upregulation of *Sox9* mRNA and SOX9 protein levels induced by KC (Fig. S4A,B).

### IL‐8 homologues upregulate *Sox9* mRNA and SOX9 protein levels in primary mouse mature hepatocytes

Next, we examined the effects of KC and MIP‐2 on *Sox9* mRNA and SOX9 protein levels in primary mouse hepatocytes. The purity of the hepatocytes was confirmed by flow cytometry which showed that isolated primary hepatocytes were over 90% pure (Fig. S5). Consistent with previous reports, isolated primary hepatocytes expressed the *Cxcr2* gene but not *Cxcr1* gene 40, 41 (Fig. S6). In these cells, MIP‐2 significantly upregulated *Sox9* mRNA levels (*P* = 0.0096) (Fig. 3E). KC also tended to upregulate *Sox9* mRNA levels, but not in a statistically significant manner. Similar to AML12 cells, SOX9‐expressing cells were observed in KC‐ or MIP‐2‐treated primary hepatocytes (Fig. 3F). EpCAM‐ or CK19‐protein was not detected in KC‐ or MIP‐2‐treated primary hepatocytes (Fig. S3B). Administration of 10 ng·mL^−1^ of MIP‐2 with 10 µm of DAPT inhibited the upregulation of *Sox9* mRNA and SOX9 protein levels induced by MIP‐2 (Fig. S4C,D).

### SOX9‐positive hepatocytes appear in the periportal area in liver biopsies of patients with acute liver injury

**Figure 4:**
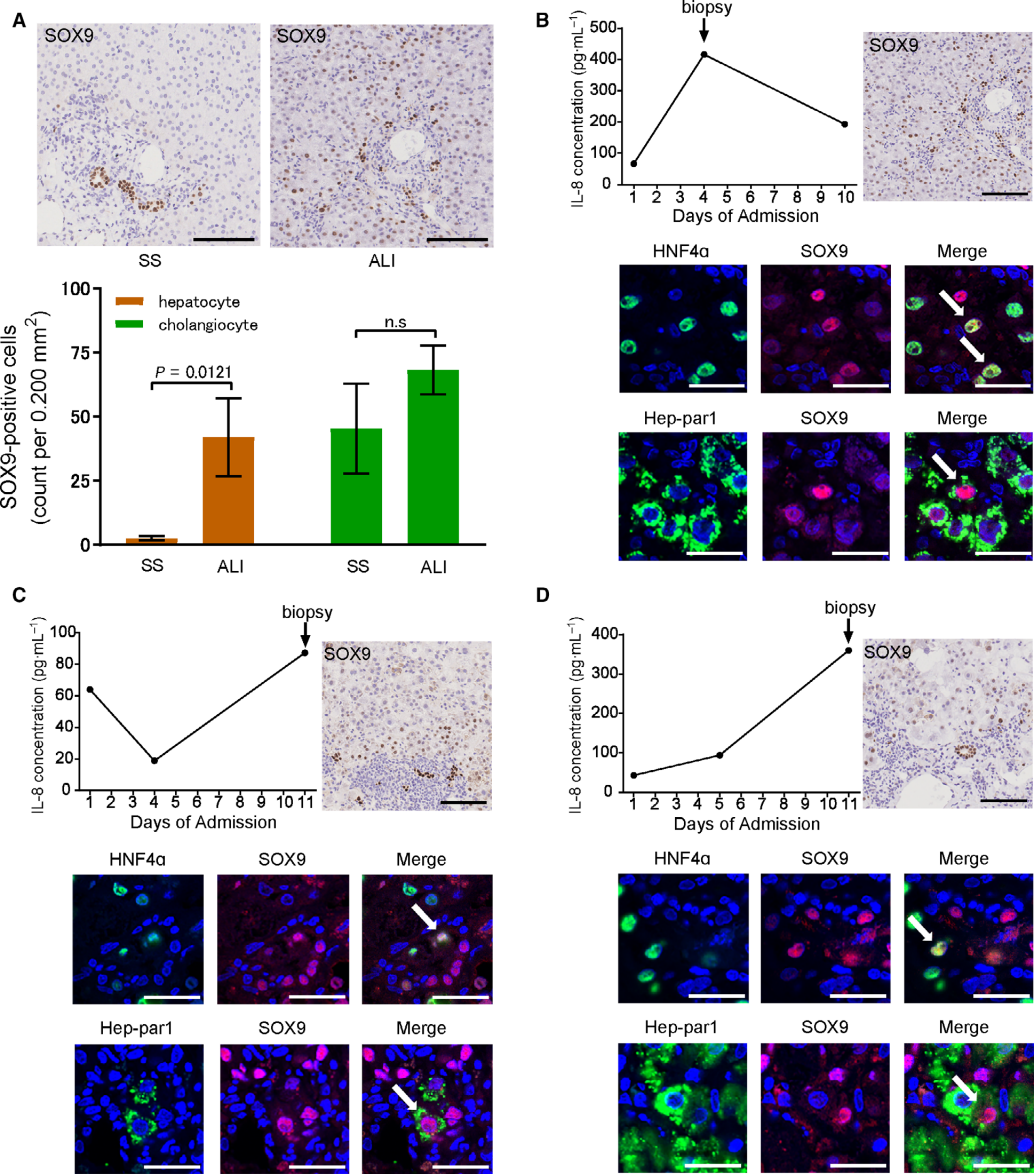
Colorimetric immunohistochemical staining and immunofluorescent detection of liver biopsy specimens. (A) SOX9 staining of liver biopsy specimens of ALI and SS patients. Mean ± SEM (SS, *n* = 3; ALI, *n* = 8). (B) A 53‐year‐old woman with autoimmune hepatitis. (C) A 35‐year‐old man with acute hepatitis A. (D) A 64‐year‐old man with acute hepatitis B. Scale bars: 100 μm for colorimetric immunohistochemical staining images and 30 µm for immunofluorescent staining images. SS, simple steatosis.

To investigate SOX9 expression in the liver tissues of acute liver injury (ALI) patients, we performed immunostaining of SOX9 in liver biopsy specimens of eight ALI patients. Because of bleeding risk associated with coagulopathy, liver biopsy of ALF patients is unfeasible during clinical practice. On the other hand, ALI patients have a low bleeding risk compared to ALF or SLI, and therefore immunostaining of liver biopsy specimens was performed in ALI patients. ALI is defined as an acute hepatic illness of < 26 weeks with a PT‐INR less than 1.5 and without preexisting chronic liver disease. Intriguingly, SOX9‐positive hepatocytes were observed in a small portion of the periportal area, as well as in preexisting cholangiocytes. It has been shown that SOX9‐positive hepatocytes are present even in normal liver tissue 21. Therefore, we used a liver biopsy specimen of a patient with simple steatosis, which is assumed to have neither bile ducts nor proliferating SOX9‐positive hepatocytes, as a control. The number of SOX9‐positive hepatocytes was significantly higher in ALI patients than in the simple steatosis patients (*P* = 0.0121) (Fig. 4A). Next, we evaluated whether SOX9‐positive cells observed in the periportal area expressed the conventional mature hepatocyte markers HNF4α and Hep‐par1. Double immunofluorescence staining of serial liver sections demonstrated that SOX9/HNF4α and SOX9/Hep‐par1 double‐positive cells were observed in the periportal area (Fig. 4B–D). We further examined the expression of EpCAM and CK19 to characterize SOX9‐positive cells. EpCAM/HNF4α or CK19/HNF4α double‐positive cells were not observed in these liver sections, and SOX9/HNF4α double‐positive cells were not coexpressed with CK19 (Fig. S7A–C). In addition, serum IL‐8 levels were elevated during the clinical course of each patient, including the timing of liver biopsy. Serum CXCL1 and CXCL2 levels were also elevated (Fig. S8). Serum IL‐8 levels were significantly higher in ALI patients than in the simple steatosis patients (*P* = 0.0485) (Fig. S9).

## Discussion

In this study, we show that, among various plasma cytokines, IL‐8 was significantly elevated in ALF and SLI patients. We also show that the IL‐8 homologues KC and MIP‐2 induced expression of SOX9, a biliary epithelial/liver progenitor cell marker, in mouse mature hepatocytes *in vitro*. Moreover, in liver biopsy specimens of ALI patients, we demonstrate that SOX9‐positive hepatocytes appeared in the liver parenchyma accompanied by elevation of IL‐8 levels. SOX9‐positive hepatocytes, a liver cell population located in the portal triads of healthy livers, undergo extensive proliferation in a murine chronic liver injury model 21. Accumulating evidence has shown that SOX9‐positive hepatocytes appear in fibrotic processes in chronic liver disease patients 42, 43. Our immunohistochemical staining results of liver biopsy specimens indicate that SOX9‐positive hepatocytes also increase in human ALI patients. An important question for the future is to determine the pathophysiological role of the increase in these cell populations.

We have previously shown that plasma of ALF patients promotes the proliferation of LPCs 22. Additionally, using our murine hepatectomy model that involves reactions to acute local injury with inflammation, we have demonstrated that immature biliary epithelial cells appear in the injury front 23. From these studies, we hypothesized that some inflammatory cytokines are substantially responsible for the induction of cells with bipotency. Recently, it has been suggested that mature hepatocytes might transdifferentiate into a cholangiocyte phenotype and be the origin of bipotential cells 13, 14, 15. In our present study, IL‐8 had the highest levels among the measured cytokines in plasma of ALF and SLI patients. We also confirmed that serum IL‐8 levels were elevated when SOX9‐positive hepatocytes were observed in the liver biopsy specimens. Therefore, IL‐8 is considered a candidate for triggering the phenotypic conversion of mature hepatocytes to cholangiocytes in plasma of ALF patients.

Few studies have focused on the relationship between cytokines and SOX9 expression in mature hepatocytes. It has been reported that TNF‐α is responsible for inducing the morphological change of hepatocytes into biliary epithelial cells *in vitro* 37. IL‐8/CXCL8 is a member of the CXC chemokine family that attracts and activates neutrophils in inflammatory regions 44. It has been reported that serum IL‐8 levels are elevated in *N*‐acetyl‐*p*‐aminophenol overdose patients and are predictive of hepatocellular damage 32. In addition, IL‐8 levels are significantly elevated in the plasma as well as in the liver of chronic liver disease patients depending on the stage of cirrhosis 33. Our *in vitro* experiments show that the IL‐8 homologues KC and MIP‐2 induce SOX9 expression in mouse mature hepatocytes. These findings support the concept that inflammatory cytokines induce phenotypic conversion of mature hepatocytes into cholangiocytes.

A previous study reported that SOX9 is a direct target of Notch signaling 20. Activation of Notch signaling in mature hepatocytes results in the conversion to a biliary epithelial cell phenotype with increasing SOX9 expression levels 15, 38, 39. Our *in vitro* experiments suggested that phenotypic conversion from hepatocytes to cholangiocytes, induced by IL‐8 homologues, is associated with Notch signaling pathways.

In conclusion, our data support the concept that immature bile duct phenotypic cells may derive from mature hepatocytes in an inflammatory environment. This molecular mechanism is expected to be conserved in human ALI. These findings may provide insight into the significance of hepatocyte plasticity during the process of liver injury as well as for elucidating the pathology of acute liver diseases.

## Conflicts of interest

The authors declare no conflict of interest.

## Author contributions

TSa carried out most experiments, analyzed the data, and wrote the manuscript. YS designed and supervised the study and wrote the manuscript. TW and HA performed primary hepatocyte isolation experiments. AS supported cell culture and immunofluorescence assays. KK performed Bio‐plex assays, cell proliferation assays, and western blotting. KI and TSu provided liver biopsy specimens. YT critically revised and finalized the manuscript.

## Supporting information


**Fig. S1.** Macroscopic view of mouse liver after perfusion with liver perfusion medium containing 4 mg/mL digitonin.
**Fig. S2.** Quantitative reverse transcription PCR of *Sox9* mRNA. AML12 cells were cultured in the absence or presence of 10 ng/mL TNF‐α. mRNA levels were normalized using 18s rRNA as a housekeeping gene. Student's unpaired two‐tailed t‐test was performed. Results are represented as mean ± s.e.m. (n = 3).
**Fig. S3.** Immunofluorescent staining of EpCAM and CK19 in mouse hepatocytes. (A) EpCAM and CK19 staining in 603B and AML12 cells treated with or without KC and MIP‐2 after 120 h of incubation. Scale bar, 100 μm. (B) EpCAM and CK19 staining in primary hepatocytes treated with or without KC and MIP‐2 after 72 h of incubation. Scale bar, 100 μm. PH, primary hepatocyte.
**Fig. S4.** Effects of Notch signaling on Sox9 mRNA and SOX9 protein levels in mouse liver cells. (A) *Sox9 *mRNA levels in AML12 cells in response to treatment with 10 ng/mL of KC, 10 μM of DAPT, and their combination. After incubation for 24 h, medium with 10 ng/mL of KC, 10 μM of DAPT, and their combination was changed at 24 h and 72 h. *Sox9 *mRNA levels in AML12 cells collected after 120 h of incubation was determined using quantitative reverse transcription PCR (qRT‐PCR). mRNA levels were normalized using 18s rRNA as a housekeeping gene. Student's unpaired two‐tailed t‐test was performed. Results are represented as mean ± s.e.m. (n = 3). (B) Immunofluorescent staining of SOX9 in AML12 cells treated with 10 ng/mL of KC, 10 μM of DAPT, and their combination after 120 h of incubation. Scale bar, 100 μm. (C) *Sox9 *mRNA levels in primary hepatocytes in response to treatment with 10 ng/mL of MIP‐2, 10 μM of DAPT, and their combination. After incubation for 24 h, medium with 10 ng/mL of MIP‐2, 10 μM of DAPT, and their combination was changed at 24 h and 48 h. *Sox9 *mRNA levels in primary hepatocytes collected after 72 h of incubation was determined using qRT‐PCR. mRNA levels were normalized using 18s rRNA as a housekeeping gene. Student's unpaired two‐tailed t‐test was performed. Results are represented as mean ± s.e.m. (n = 3). (D) Immunofluorescent staining of SOX9 in primary hepatocytes treated with 10 ng/mL of MIP‐2, 10 μM of DAPT, and their combination after 72 h of incubation. Scale bar, 100 μm. PH, primary hepatocyte.
**Fig. S5.** Flow cytometric analysis of primary mouse hepatocyte purity.
**Fig. S6.** *Cxcr1* and *Cxcr2* mRNA levels in primary mouse hepatocytes. *Gapdh* was used as the internal control.
**Fig. S7.** Immunofluorescent detection of liver biopsy specimens. (A) A 53‐year‐old woman with autoimmune hepatitis. (B) A 35‐year‐old man with acute hepatitis A. (C) A 64‐year‐old man with acute hepatitis B. Scale bars: 50 μm for immunofluorescent staining images. Arrows indicate SOX9/HNF4α double positive cells.
**Fig. S8.** Serum CXCL1 and CXCL2 levels in acute liver injury patients. (A) A 53‐year‐old woman with autoimmune hepatitis. (B) A 35‐year‐old man with acute hepatitis A. (C) A 64‐year‐old man with acute hepatitis B.
**Fig. S9.** Serum interleukin (IL)‐8 levels of simple steatosis and acute liver injury patients. Mann‐Whitney *U*‐test was performed. Results are represented as mean ± s.e.m. (SS, n = 3; ALI, n = 8) SS, simple steatosis. ALI, acute liver injury.
**Table S1.** Definition of ALF, SLI and ALI patients.
**Table S2.** Primers used for semi‐quantitative RT‐PCR analysis.
**Table S3.** Primers used for quantitative real‐time PCR analysis.
**Table S4.** Serum cytokine levels in ALF and SLI patients.
