# Supplementary material for: IL‐8 induces transdifferentiation of mature hepatocytes toward the cholangiocyte phenotype
Source: FEBS Open Bio. 2019 Nov 7;9(12):2105–16. doi: 10.1002/2211-5463.12750 (PMC6886300; doi:10.1002/2211-5463.12750)

Supplementary Information

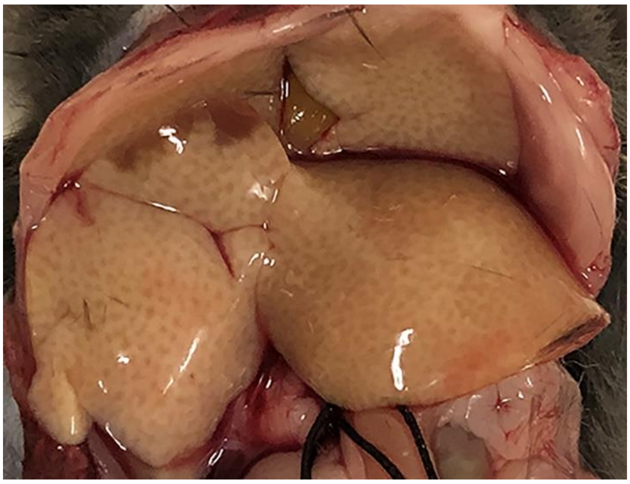

Supplementary Figure 1. Macroscopic view of mouse liver after perfusion with liver perfusion medium containing 4 mg/mL digitonin.

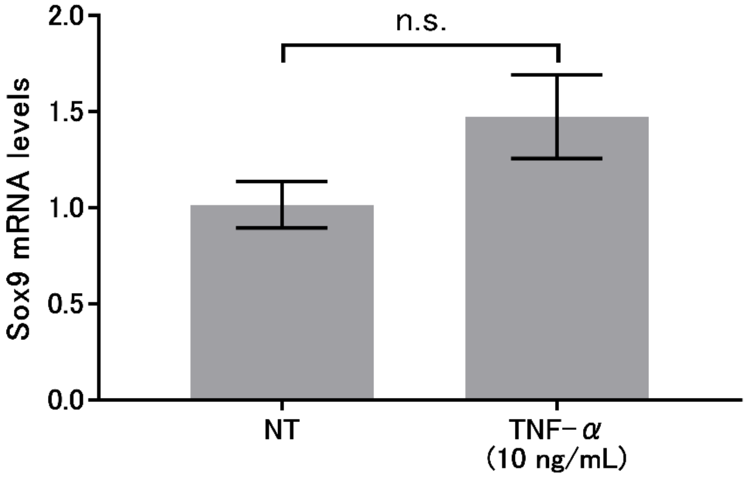

Supplementary Figure 2. Quantitative reverse transcription PCR of *Sox9* mRNA. AML12 cells were cultured in the absence or presence of 10 ng/mL TNF-α. mRNA levels were normalized using 18s rRNA as a housekeeping gene. Student's unpaired two-tailed *t*-test was performed. Results are represented as mean ± s.e.m. (n = 3).

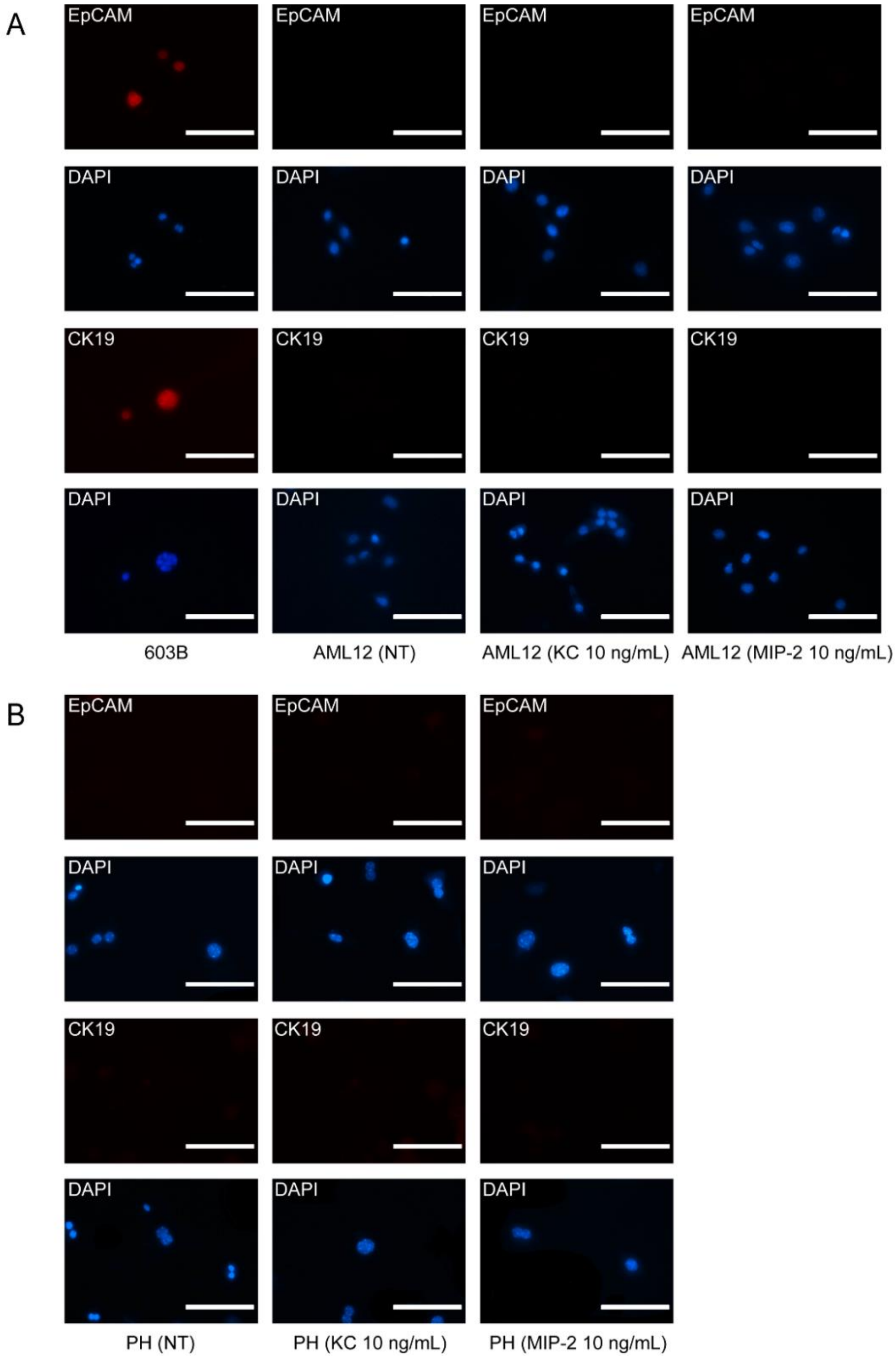

Supplementary Figure 3. Immunofluorescent staining of EpCAM and CK19 in mouse hepatocytes. (A) EpCAM and CK19 staining in 603B and AML12 cells treated with or without KC and MIP-2 after 120 h of incubation. Scale bar, 100 μm. (B) EpCAM and CK19 staining in primary hepatocytes treated with or without KC and MIP-2 after 72 h of incubation. Scale bar, 100 μm. PH, primary hepatocyte.

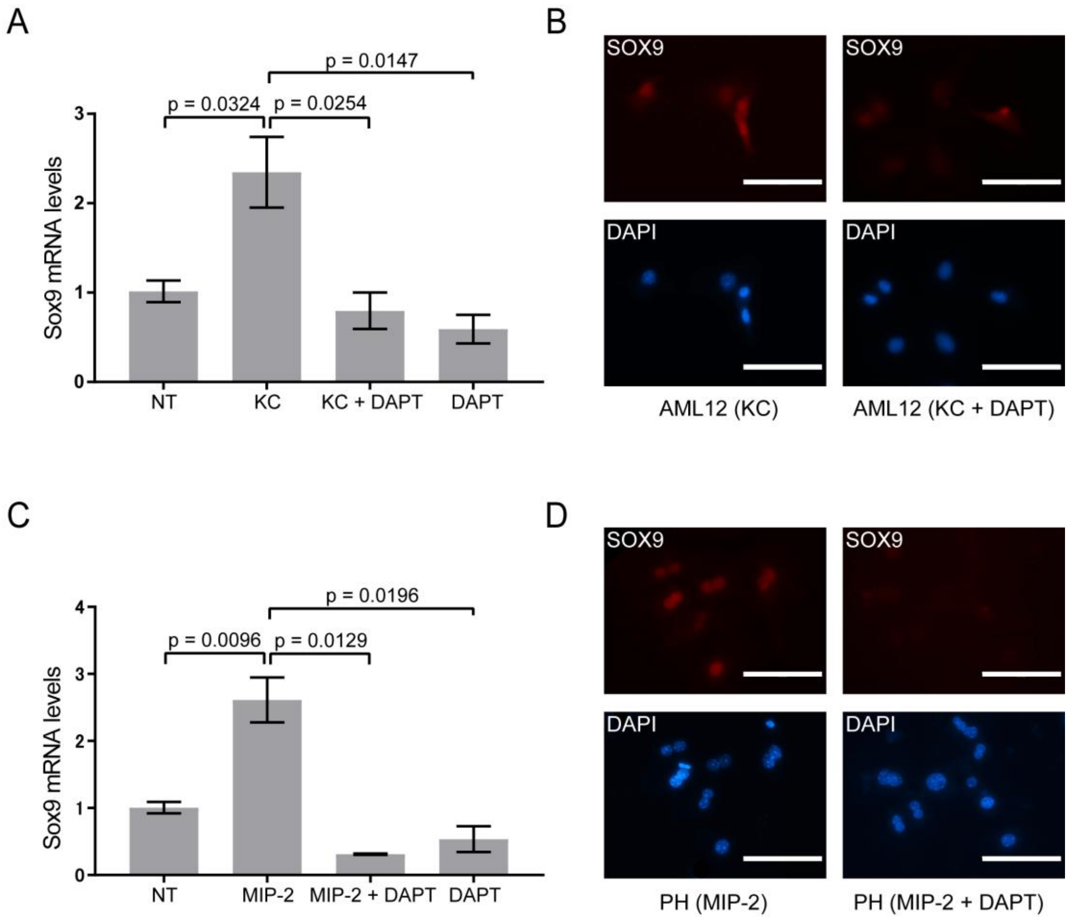

Supplementary Figure 4. Effects of Notch signaling on *Sox9* mRNA and SOX9 protein levels in mouse liver cells. (A) *Sox9* mRNA levels in AML12 cells in response to treatment with 10 ng/ml of KC, 10 μM of DAPT, and their combination. After incubation for 24 h, medium with 10 ng/mL of KC, 10 μM of DAPT, and their combination was changed at 24 h and 72 h. *Sox9* mRNA levels in AML12 cells collected after 120 h of incubation was determined using quantitative reverse transcription PCR (qRT-PCR). mRNA levels were normalized using 18s rRNA as a housekeeping gene. Student's unpaired two-tailed t-test was performed. Results are represented as mean ± s.e.m. (n = 3). (B) Immunofluorescent staining of SOX9 in AML12 cells treated with 10 ng/ml of KC, 10 μM of DAPT, and their combination after 120 h of incubation. Scale bar, 100 μm. (C) *Sox9* mRNA levels in primary hepatocytes in response to treatment with 10 ng/ml of MIP-2, 10 μM of DAPT, and their combination. After incubation for 24 h, medium with 10 ng/mL of MIP-2, 10 μM of

DAPT, and their combination was changed at 24 h and 48 h. *Sox9* mRNA levels in primary hepatocytes collected after 72 h of incubation was determined using qRT-PCR. mRNA levels were normalized using 18s rRNA as a housekeeping gene. Student's unpaired two-tailed t-test was performed. Results are represented as mean ± s.e.m. (n = 3). (D) Immunofluorescent staining of SOX9 in primary hepatocytes treated with 10 ng/ml of MIP-2, 10 μM of DAPT, and their combination after 72 h of incubation. Scale bar, 100 μm. PH, primary hepatocyte.

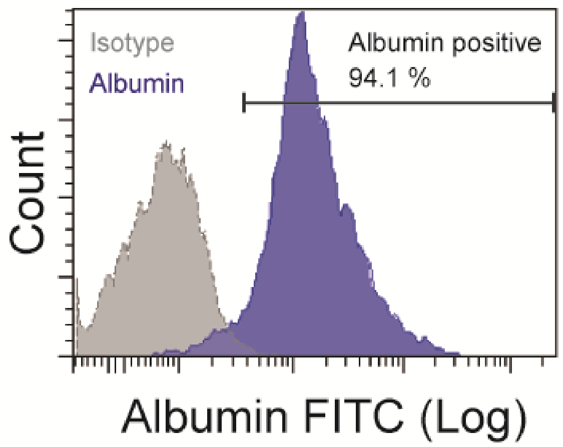

Supplementary Figure 5. Flow cytometric analysis of primary mouse hepatocyte purity.

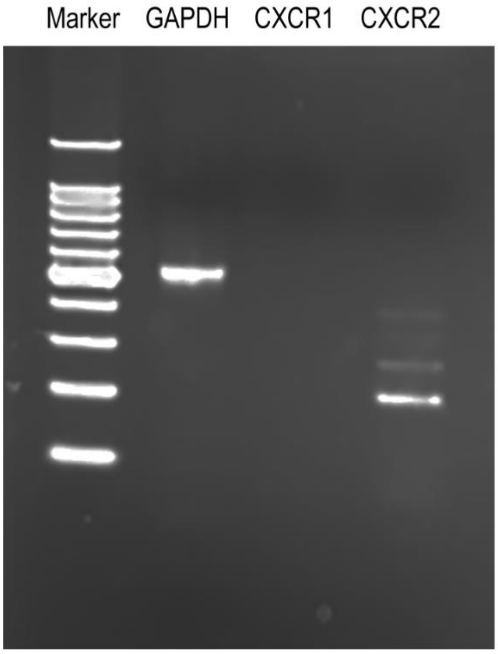

Supplementary Figure 6. *Cxcr1* and *Cxcr2* mRNA levels in primary mouse hepatocytes. *Gapdh* was used as the internal control.

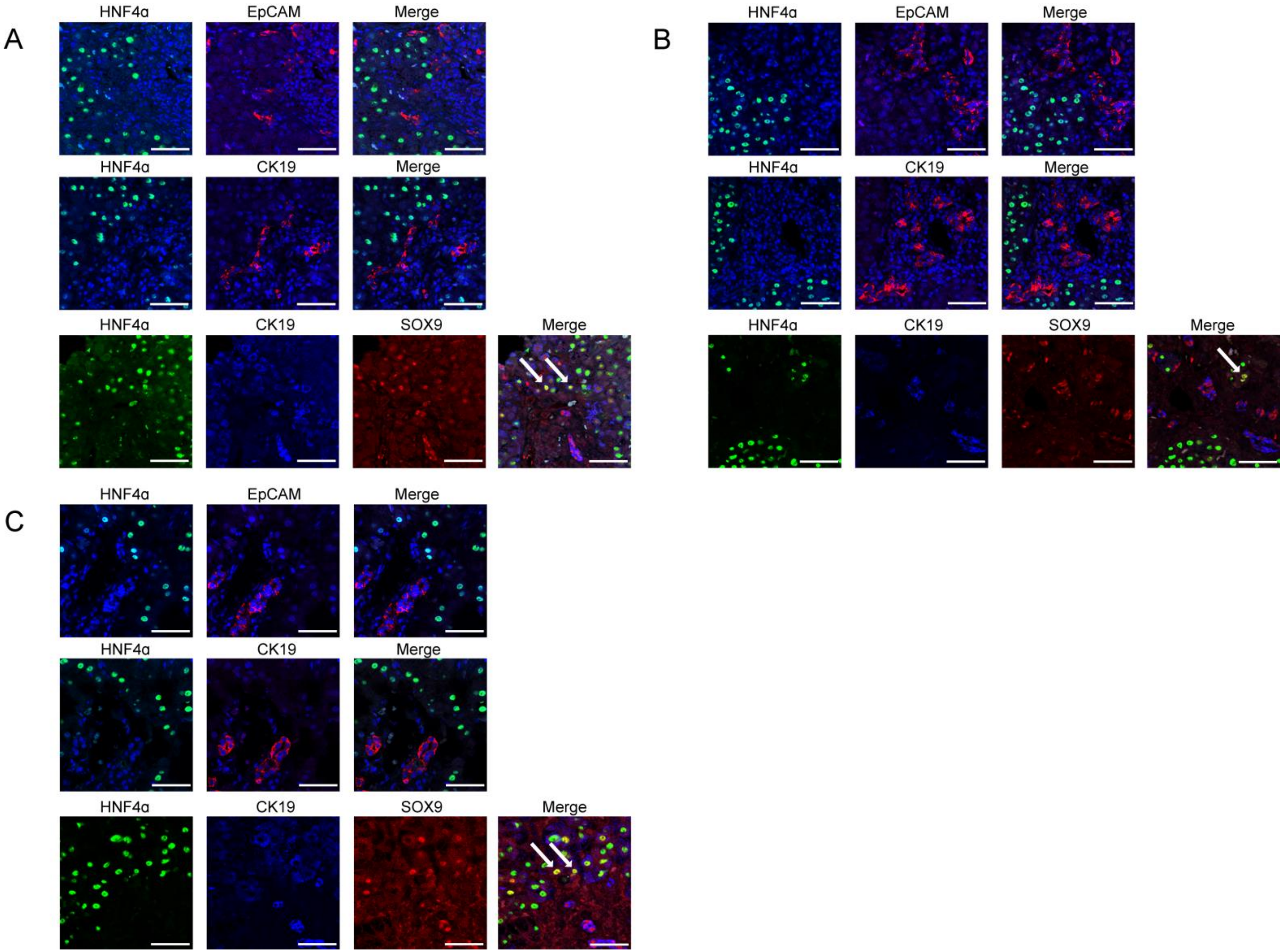
A
HNF4α
EpCAM
Merge
HNF4α
CK19
Merge
HNF4α
CK19
SOX9
Merge
B
HNF4α
EpCAM
Merge
HNF4α
CK19
Merge
HNF4α
CK19
SOX9
Merge
C
HNF4α
EpCAM
Merge
HNF4α
CK19
Merge
HNF4α
CK19
SOX9
Merge

Supplementary Figure 7. Immunofluorescent detection of liver biopsy specimens. (A) A 53-year-old woman with autoimmune hepatitis. (B) A 35-year-old man with acute hepatitis A. (C) A 64-year-old man with acute hepatitis B. Scale bars: 50 μm for immunofluorescent staining images. Arrows indicate SOX9/HNF4α double positive cells.

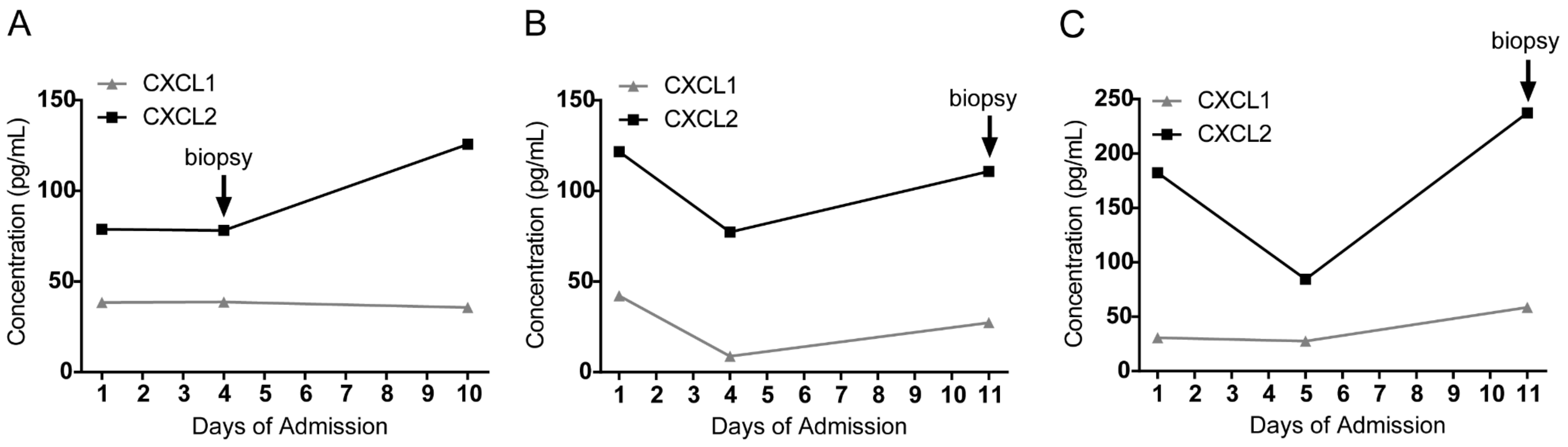

Supplementary Figure 8. Serum CXCL1 and CXCL2 levels in acute liver injury patients. (A) A 53-year-old woman with autoimmune hepatitis. (B) A 35-year-old man with acute hepatitis A. (C) A 64-year-old man with acute hepatitis B.

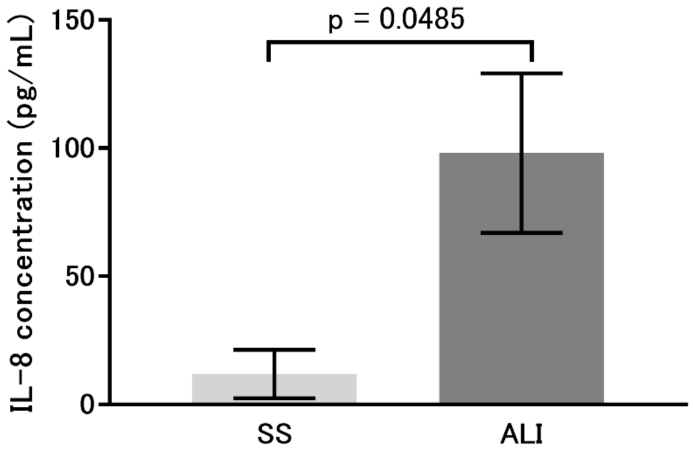

Supplementary Figure 9. Serum interleukin (IL)-8 levels of simple steatosis and acute liver injury patients. Mann-Whitney *U*-test was performed. Results are represented as mean ± s.e.m. (SS, n = 3; ALI, n = 8) SS, simple steatosis. ALI, acute liver injury.

Supplementary Table 1. Definition of ALF, SLI and ALI patients

	Definition
ALF	Acute hepatic illness of < 26 weeks with a PT-INR of ≥ 1.5 and any degree of hepatic encephalopathy in a patient without pre-existing chronic liver disease.
SLI	Acute hepatic illness of < 26 weeks with a PT-INR of ≥ 1.5, along with absence of hepatic encephalopathy in a patient without pre-existing chronic liver disease.
ALI	Acute hepatic illness of < 26 weeks with a PT-INR of < 1.5 in a patient without pre-existing chronic liver disease.

ALF, acute liver failure; SLI, severe acute liver injury; ALI, acute liver injury; PT-INR, prothrombin time-international normalized ratio.

Supplementary Table 2. Primers used for semi-quantitative RT-PCR analysis

Gene name	Forward primer (5` to 3`)	Reverse primer (5` to 3`)
**Cxcr1**	**CGCTGGTGATGCTGGTTATC**	**TAGGGCAGACAACAGAGCAA**
**Cxcr2**	**GGTGGGGAGTTCGTGTAGAA**	**CGAGGTGCTAGGATTTGAGC**
**Gapdh**	**ATTCAACGGCACAGTCAAGG**	**TGGATGCAGGGATGATGTTC**

Supplementary Table 3. Primers used for quantitative real-time PCR analysis

Gene name	Forward primer (5` to 3`)	Reverse primer (5` to 3`)
**Sox9**	**CAGCAAGACTCTGGGCAAG**	**ATCGGGGTGGTCTTTCTTGT**
**18s**	**GCAATTATTCCCCATGAACG**	**GGGACTTAATCAACGCAAGC**

Supplementary Table 4. Serum cytokine levels in ALF and SLI patients

	Normal range (pg/mL)	ALF and SLI n = 37
IL-1β	< 2.0	24.5 (14.5-31.5)
IL-4	< 2.0	23.5 (13.5-37.0)
IL-5	< 2.0	21.9 (9.5-35.5)
IL-6	< 2.0	58.5 (42.5-109.5)
IL-7	< 14.5	30.0 (18.5-41.5)
IL-8	< 2.0	111.0 (84.0-140.0)
G-CSF	< 2.0	24.3 (11.3-37.3)
IFN-γ	< 2.0	25.0 (13.0-40.0)
TNF-α	< 2.0	19.5 (9.5-27.5)

ALF, acute liver failure; SLI, severe acute liver injury.

Unedited gels for Figure 2A: images cropped to dotted-line boxes.

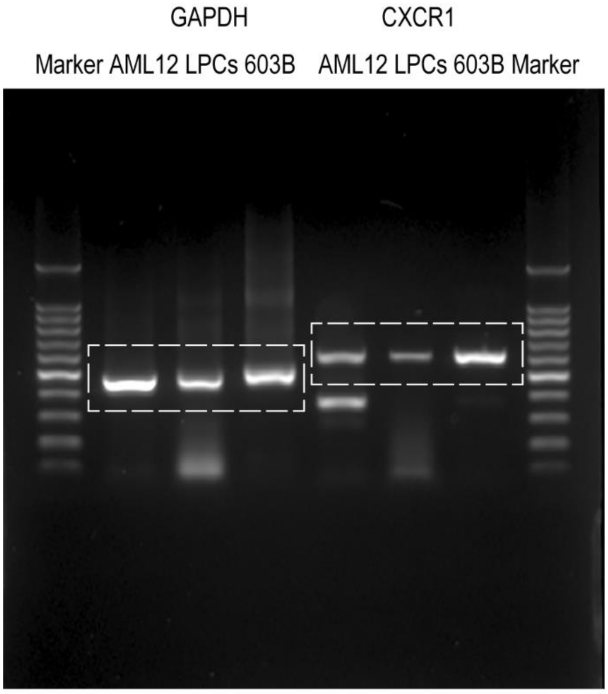

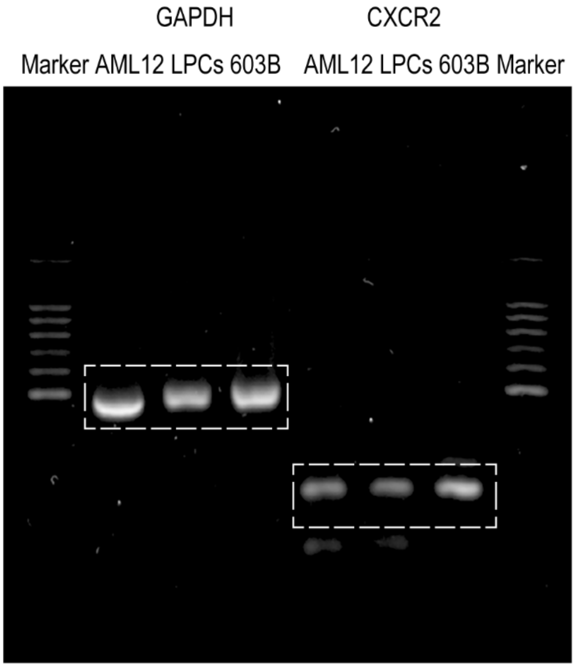

Unedited blots for Figure 2C: images cropped to dotted-line boxes.

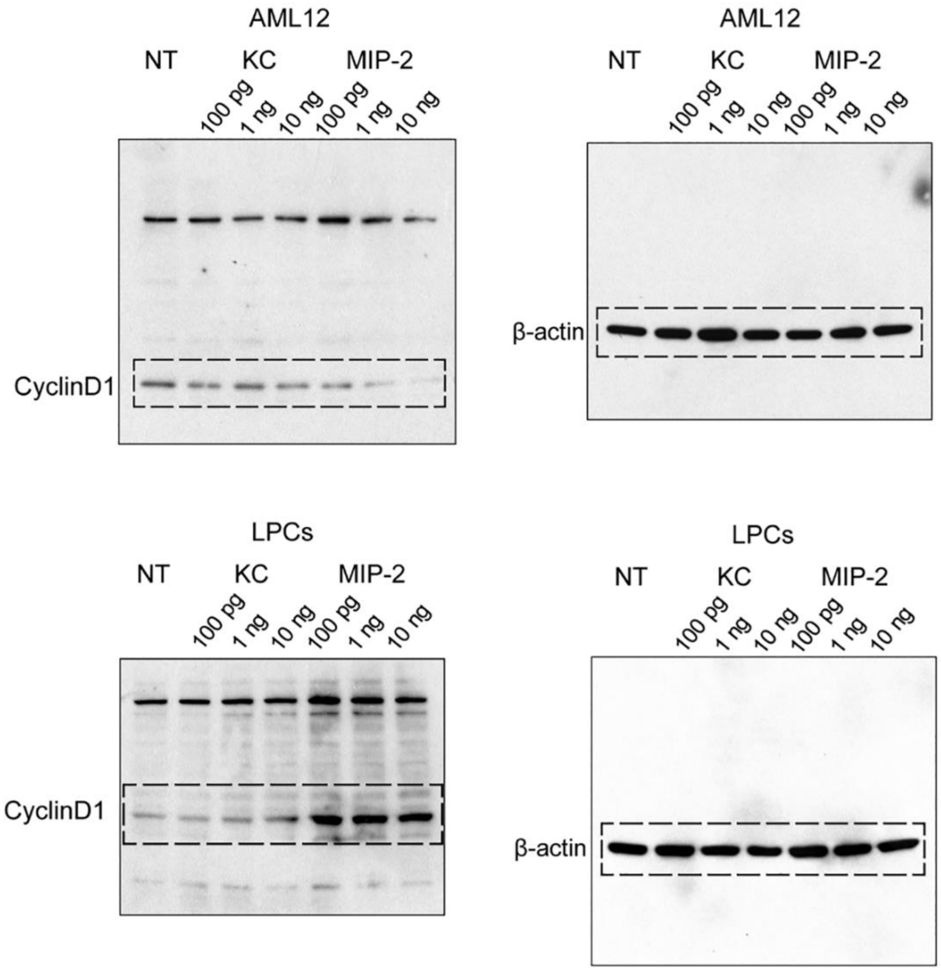

Supplement: Supplementary file 1 — Fig. S1. Macroscopic view of mouse liver after perfusion with liver perfusion medium containing 4 mg/mL digitonin. Fig. S2. Quantitative reverse transcription PCR of Sox9 mRNA. AML12 cells were cultured in the absence or presence of 10 ng/mL TNF‐α. mRNA levels were normalized using 18s rRNA as a housekeeping gene. Student's unpaired two‐tailed t‐test was performed. Results are represented as mean ± s.e.m. (n = 3). Fig. S3. Immunofluorescent staining of EpCAM and CK19 in mouse hepatocytes. (A) EpCAM and CK19 staining in 603B and AML12 cells treated with or without KC and MIP‐2 after 120 h of incubation. Scale bar, 100 μm. (B) EpCAM and CK19 staining in primary hepatocytes treated with or without KC and MIP‐2 after 72 h of incubation. Scale bar, 100 μm. PH, primary hepatocyte. Fig. S4. Effects of Notch signaling on Sox9 mRNA and SOX9 protein levels in mouse liver cells. (A) Sox9 mRNA levels in AML12 cells in response to treatment with 10 ng/mL of KC, 10 μM of DAPT, and their combination. After incubation for 24 h, medium with 10 ng/mL of KC, 10 μM of DAPT, and their combination was changed at 24 h and 72 h. Sox9 mRNA levels in AML12 cells collected after 120 h of incubation was determined using quantitative reverse transcription PCR (qRT‐PCR). mRNA levels were normalized using 18s rRNA as a housekeeping gene. Student's unpaired two‐tailed t‐test was performed. Results are represented as mean ± s.e.m. (n = 3). (B) Immunofluorescent staining of SOX9 in AML12 cells treated with 10 ng/mL of KC, 10 μM of DAPT, and their combination after 120 h of incubation. Scale bar, 100 μm. (C) Sox9 mRNA levels in primary hepatocytes in response to treatment with 10 ng/mL of MIP‐2, 10 μM of DAPT, and their combination. After incubation for 24 h, medium with 10 ng/mL of MIP‐2, 10 μM of DAPT, and their combination was changed at 24 h and 48 h. Sox9 mRNA levels in primary hepatocytes collected after 72 h of incubation was determined using qRT‐PCR. mRNA levels were normalized using 18s rRNA [file FEB4-9-2105-s001.pdf]
